# Future prospects of immune checkpoint blockade in cancer: from response prediction to overcoming resistance

**DOI:** 10.1038/s12276-018-0130-1

**Published:** 2018-08-22

**Authors:** Young-Jun Park, Da-Sol Kuen, Yeonseok Chung

**Affiliations:** 1 Laboratory of Immune Regulation, Research Institute of Pharmaceutical Sciences, College of Pharmacy, Seoul National University, Seoul, 08826 Republic of Korea; 2 BK21 Plus program, College of Pharmacy, Seoul National University, Seoul, 08826 Republic of Korea

## Abstract

Recent advances in the understating of tumor immunology suggest that cancer immunotherapy is an effective treatment against various types of cancer. In particular, the remarkable successes of immune checkpoint-blocking antibodies in clinical settings have encouraged researchers to focus on developing other various immunologic strategies to combat cancer. However, such immunotherapies still face difficulties in controlling malignancy in many patients due to the heterogeneity of both tumors and individual patients. Here, we discuss how tumor-intrinsic cues, tumor environmental metabolites, and host-derived immune cells might impact the efficacy and resistance often seen during immune checkpoint blockade treatment. Furthermore, we introduce biomarkers identified from human and mouse models that predict clinical benefits for immune checkpoint blockers in cancer.

## Cancer immunotherapy: Boosting anti-tumor immunity

New immunotherapies for cancer known as immune checkpoint blockers (ICBs) may help treat many tumor types. Homeostasis of the immune system is controlled by proteins known as immune checkpoints; cancer cells evade the anti-tumor immune response by displaying ligands of the immune checkpoint molecules, thereby incapacitating tumor-killing immune cells. ICBs disable the checkpoints, making cancer cells vulnerable to the immune response. Although ICBs are effective against many cancers, some patients show resistance or relapse. In a review of ICBs, Young-Jun Park and Yeonseok Chung at Seoul National University, South Korea, report that combining two ICBs can improve efficacy, and discuss new pre-, and post-treatment tests to determine whether a drug will work for a particular patient. They also report that manipulating patients’ intestinal microflora can improve response to ICBs. The findings reported may help to optimize these promising treatments.

## Introduction

Historical records suggest that scientists have sought for over a century to activate and harness the body’s immune response in order to eradicate cancerous cells. It was first attempted in 1891, based on observations regarding the remissions of tumors after surgery in patients with infections^1^. To reproduce this phenomenon, William Coley attempted to inject inactivated or live *Streptococcus pyogenes* organisms into a patient with neck and tonsil cancer. Due to bacterial-induced inflammation, the patient developed a high fever; however, the tumor burden regressed. Oncologists at the time assumed that the bystander killing effect of inflammation reduced tumor size but disregarded Coley’s approach due to the lack of exact scientific proof and risks concerning the inoculation of infectious organisms. By the 1990s, however, the development of mouse systems of pure genetic background enabled researchers to revisit the cancer immune-surveillance theory and to elucidate how the tumor environment is sculpted by the immune system to eventually either be eliminated or ignored^2–4^.

Despite decades of bench-side research, it has only been several years since immunotherapy was allowed to move into the mainstream of cancer therapeutics in the clinic. Recent approvals by the US Food and Drug Administration (FDA) of ipilimumab, a cytotoxic T lymphocyte-associated protein 4 (CTLA-4) blocking antibody, for the treatment of melanoma^5^ has encouraged the placement of immunotherapy at the forefront of cancer treatment. CTLA-4 was first known as a member of the immunoglobulin superfamily induced by activated T cells to transmit self-inhibitory signals^6^. Subsequently, antibodies blocking the programmed cell death protein 1 (PD-1):PD-L1 pathway, now referred to as an immune checkpoint, along with CTLA-4, have demonstrated promising effects in patients for treating more than ten types of cancers, including non-small-cell lung carcinoma (NSCLC) and renal cell carcinoma (RCC)^7–9^. Soon after, additional immune checkpoints were discovered, leading researchers to focus on the development of new-generations of immune checkpoint blockers. However, numerous populations of cancer patients remain uncured by these treatments, necessitating novel therapeutic solutions for non-responders.

In this review, we summarize the current status of immune checkpoint blockers in clinical settings and discuss the efficacy of applying several immune checkpoint-blocking antibodies in combination. We also introduce comprehensive clinical studies that identify biomarkers for distinguishing responsive from non-responsive or resistant cancers in patients treated with immune checkpoint blockers. We also discuss how regulating tumor-autonomous factors is critical for immuno-resistance and how using agents that control tumor-extrinsic factors influence anti-tumor immunity.

## Immune checkpoint blockers (ICBs)

Immune checkpoints consist of various inhibitory pathways that act as homeostatic regulators of the immune system and are crucial for maintaining central/peripheral tolerance as well as reducing excessive systemic inflammation in the body^10^. In the tumor environment, however, tumors hijack these inhibitory mechanisms to avoid anti-tumor immune responses.

### CTLA-4

**Fig. 1:**
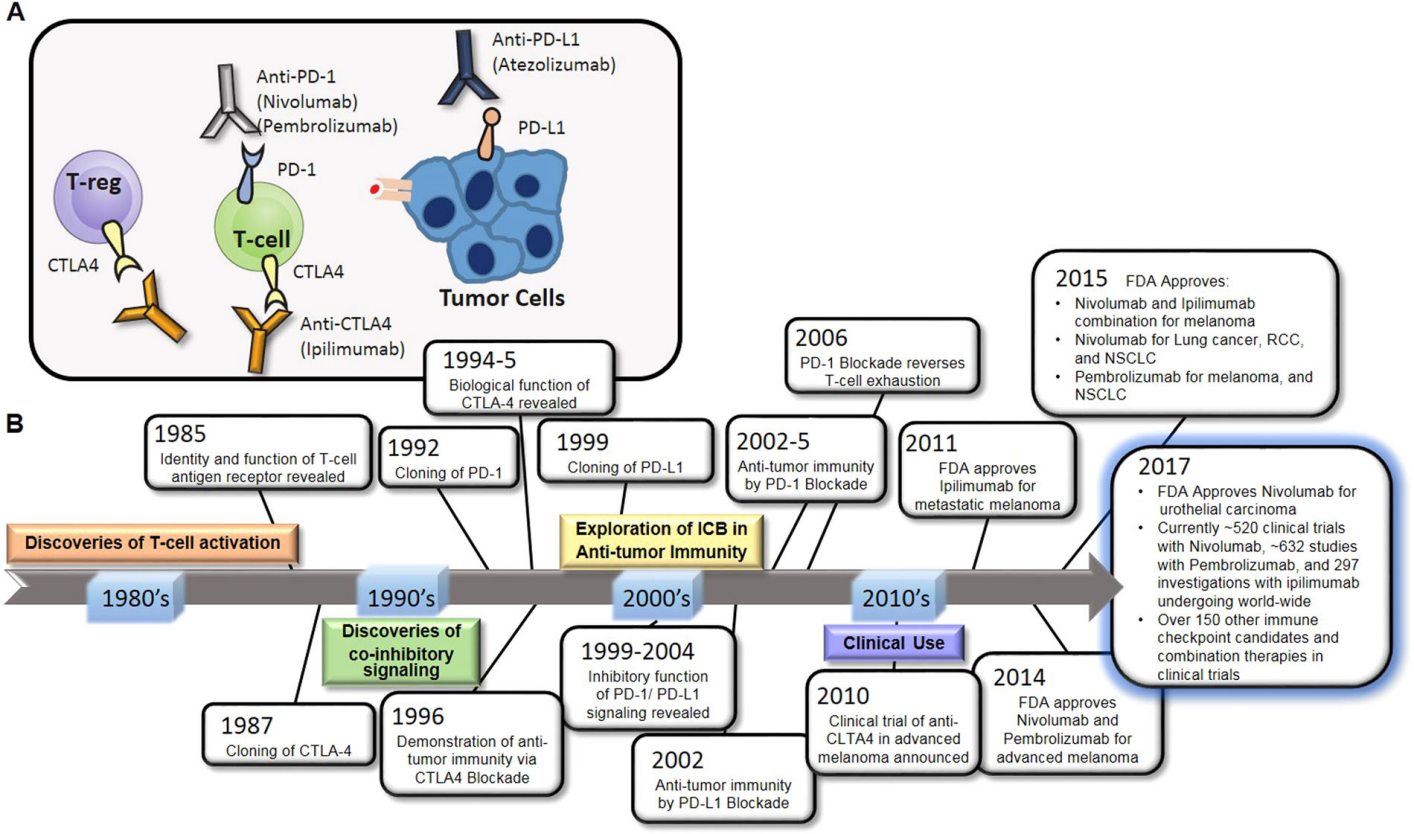
Timeline highlights of ICB therapy development within the last three decades. **a** Schematic of the mechanism of action of ICB agents. **b** Timeline highlights of ICB therapy development from its inspection of T-cell activation mechanisms, including the discovery of CTLA-4 and PD-1/PD-L1, to recent clinical trials that are either already approved or are expected to be approved by the FDA

The first clinical development of a CTLA-4-blocking antibody, ipilimumab, proved that immune checkpoints would be attractive targets for cancer therapy. CTLA-4 is known to be expressed by activated T cells and regulatory T cells (Tregs). Together with TCR-mediated signal 1, CD28 ligation with CD80/86 on antigen-presenting cells (APCs) delivers signal 2 to trigger T-cell survival and expansion by inducing interleukin (IL)-2 production^11^. CTLA-4 binds CD80 and CD86 with a far higher affinity than CD28, thereby outcompeting for the same ligands and inhibiting TCR signaling^6,12^. As a result, CTLA-4-blocking antibodies augment the binding of CD80/86 to CD28 rather than to CTLA-4 and also deplete Tregs in the tumor environment that consistently express CTLA-4 (Fig. 1a)^13,14^.

Once the CTLA-4 signal was shown to restrict the activity of T cells, agents that shut down this signaling molecule became candidates for combination treatment with existing cancer therapeutics^15,16^. In 2010, a surprising result came from a phase III clinical study of the GP100 peptide vaccine with an anti-CTLA-4 mAb, ipilimumab (MDX-010, Bristol-Myers Squibb). Unexpectedly, only ipilimumab-treated patients showed prolonged survival compared with patients treated with the peptide vaccine alone or with the vaccine combined with ipilimumab^17^. These clinical outcomes allowed the FDA to approve ipilimumab for the treatment of melanoma in humans and led to additional approvals for the treatment of RCC^18^. Additionally, phase II and III studies showed that anti-CTLA-4 blockade treatment in advanced-melanoma patients resulted in a 22% 3-year survival rate and durable responses extending beyond 10 years^19^.

### PD-1 and PD-L1

PD-1, another member of the inhibitory receptor family, is also expressed on T cells during TCR stimulation. The expression of its ligands, PD-L1 and PD-L2, is regulated by inflammatory cytokines. While PD-L2 is exclusively induced on APCs, PD-L1 is expressed on tumor cells, epithelial cells, and immune cells. These molecules inhibit TCR downstream signaling when ligated with PD-1^20,21^ (Fig. 1a). Two different PD-1-blocking antibodies, pembrolizumab and nivolumab, are currently the most promising qualifiers expected to provide significant clinical benefit. Several publications showed that patients with advanced melanoma, NSCLC and RCC experienced objective responses following PD-1 blockade^22–24^. These results encouraged the FDA to approve those antibodies for such indications. The success of PD-1-blocking antibodies in clinical settings promoted the development of PD-L1 blockers, such as atezolizumab, for the treatment of bladder cancer patients^25^. Together with PD-1, PD-L1 binds to CD80 expressed on T-cell surfaces^26^. Due to the complicated interaction, PD-L1 still can inhibit T-cell responses during PD-1 blockade. Furthermore, PD-L1 expression has been shown to increase when tumor cells are exposed to interferon (IFN)-γ during therapy, indicating that PD-L1 blockade might have an advantage over the PD-1-blocking antibody in some cases^27,28^.

As to the novel mechanistic explanation for the immune responses that lead to anti-PD-1 blockade-mediated tumor rejection, recent studies have shown that anti-PD-1 therapy leads to a dynamic expansion and proliferation of PD-1^+^ (exhausted-like) CD8 T cells in the PBMCs of melanoma and lung cancer patients^29,30^. Despite such clinical data, it is still yet unknown whether the expansion of ICB-responsive exhausted-like CD8 T cells is driven by the direct therapeutic engagement of peripheral or tumor-infiltrating populations or what functionally distinguishes the ICB-responsive from the non-responsive exhausted-like CD8 T cells. However, chronic viral infection models suggest that it is possible that expansion of specific tumor-infiltrating PD-1^+^ CD8 T-cell subsets in response to ICB results from the selective expansion of a distinct progenitor CD8 T-cell population in the secondary lymphoid organs^31^.

### Other inhibitory receptors or combination therapy

Recent studies have suggested that several proteins expressed on T cells regulate exhaustion. Lymphocyte-activation gene 3 (LAG-3) induced on activated T cells and Tregs binds to MHC class II or galectin-3, which transduces an inhibitory signal in T cells or enhances the suppressive activity of Tregs^32,33^. Agents blocking LAG-3 are being tested in clinical trials against multiple cancers.

T-cell immunoglobulin and mucin domain-containing 3 (TIM-3; HAVCR2) is another cell surface molecule involved in T-cell exhaustion^34^. It was first demonstrated that TIM-3 controls T-cell unresponsiveness in chronic inflammation. Upon interaction with galectin-9 or other undefined ligands, TIM-3-expressing T cells undergo apoptosis and lose effector functions^35^. Further analysis showed that TIM-3 expression was upregulated in tumor-infiltrating lymphocytes (TILs) in melanoma and NSCLC patients^36,37^.

T-cell immune receptor with immunoglobulin and ITIM domain (TIGIT) is also implicated in the inhibition of T-cell activation. TIGIT expression is tightly regulated in lymphocytes, especially in Tregs and CD8 T cells^38^, and is a phenotypic marker of inert CD8 T cells or mediates Treg suppression of other effector cells^39,40^. As such, blockade of TIGIT might be considered as an attractive target in conjunction with the CTLA-4/PD-1 pathways. The current status of the development and approval of immunotherapeutic agents is listed in Table 1.

**Table 1:** Current status of immune checkpoint blockers

Target	Agent	Manufacturer	Cancer type	Stage
CTLA-4	Ipilimumab	Bristol-Myers Squibb	Melanoma	FDA-approved
			Many cancers	Phase I–III
	Tremelimumab	AstraZeneca	Melanoma, liver, mesothelioma, colorectal, lung	Phase I–III
PD-1	Nivolumab	Bristol-Myers Squibb	Melanoma, lung	FDA-approved
			Many cancers	Phase I–III
	Pembrolizumab	Merck	Melanoma	FDA-approved
			Many cancers	Phase I–III
	Pidilizumab	CureTech, Ltd	Melanoma, renal, pancreatic, prostate, lymphoma, etc	Phase I–II
	AMP-224	Amplimmune, GSK	Many cancers	Phase I
	MEDI0680	MedImmune	Many cancers	Phase I–II
PD-L1	Atezolizumab	Genentech, Roche	Bladder	FDA-approved
			Many cancers	Phase I–III
	MDX1105	Bristol-Myers Squibb	Many cancers	Phase I
	MEDI4736	Medlmmune LLC AstraZeneca	Many cancers	Phase I–III
	Avelumab	Merck, Pfizer	Many cancers	Phase I–III
LAG-3	IMP321	Immutep	Melanoma, breast, renal, pancreatic	Phase I–II
	BMS-986016	Bristol-Myers Squibb	Many cancers	Phase I–II
IDO	Epacadostat	Incyte Corporation	Melanoma, ovarian, peritoneal carcinoma, myelodysplastic syndromes	Phase I–III
	Indoximod	NewLink Genetics Corporation	Many cancers	Phase I–II
	Gdc-0919	Genentech, Roche	Many cancers	Phase I
TIM-3	TSR-022	Tesaro, Inc.	Advanced solid tumor	Phase I
	LY3321367	Eli Lilly and Co.	Solid tumor	Phase I
	MBG453	Novartis	Advanced malignancies	Phase I
TIGIT	OMP-313M32	Oncomed Pharmaceuticals, Inc.	Locally advanced, and metastatic Cancer	Phase I
	MTIG7192A	Genentech, Inc.	Solid tumors	Phase I
	BMS-986207	Bristol-Myers Squibb	Advanced solid tumors	Phase I–II
	MK-7684	Merck	Advanced solid tumors	Phase I
CD73	CPI-006	Corvus Pharmacueticals, Inc.	NSCLC, RCC, CRC, TNBC, cervical, ovarian etc.	Phase I
	MEDI9447	Medlmmune, LLC	Solid Tumors	Phase I

To improve therapeutic efficacy, a number of preclinical and clinical studies have been conducted to examine if combination treatment of immune checkpoint blockers with conventional treatments, such as chemotherapies, targeted therapies, radiation therapies, and other immunotherapeutic agents, could improve outcomes. So far, the most favorable prognosis has been the combination of CTLA-4 and PD-1-blocking antibodies. In the first clinical study performed to determine the antibody combination dose, objective response rates were exhibited in over 40% of patients across all doses^41^. Notably, this study demonstrated that 28% of patients with clinical benefits showed 80% or greater tumor regression^24^. In a sequential phase II study, 61% of the patients who received the combination therapy exhibited objective responses compared to the 11% of those treated with ipilimumab alone^42^. Although not statically significant in comparison with the PD-1 single blockade group (nivolumab), the progression-free survival (PFS) rate was clearly prolonged in the combination group compared to that of either monotherapy group (2.9%, 6.9%, and 11.5% for ipilimumab, nivolumab, and combination therapy, respectively)^43,44^. Up-to-date clinically tested combination therapies and ICB development time-lines are summarized in Table 2 and Fig. 1b, respectively^17,42,45–47^.

**Table 2:** Clinical outcomes in combination immunotherapy regimens

Agent	Treatment	Indication	Overall response (complete + partial response)
Ipilimumab and nivolumab	Nivolumab only vs. nivolumab + ipilimumab vs. ipilimumab only	Advanced-stage untreated melanoma	• 44% nivolumab • 58% ipilimumab + nivolumab • 19% ipilimumab
Ipilimumab and nivolumab	Concurrent combination with dose elevation	Advanced-stage melanoma	• 42%
Ipilimumab and nivolumab	Ipilimumab + nivolumab vs. ipilimumab only	Advanced-stage untreated melanoma	• 61% ipilimumab + nivolumab • 11% ipilimumab
Ipilimumab and bevacizumab	Concurrent combination with dose elevation	Advanced-stage melanoma	• 19.6%
Ipilimumab and GP100 vaccine	Ipilimumab only vs. ipilimumab + vaccine vs. vaccine only	Previously treated advanced-stage melanoma	• 10.9% ipilimumab only • 5.7% ipilimumab + vaccine • 1.5% vaccine only

## Clinical prognosis indicators for immune checkpoint blockers: responder or non-responder?

**Fig. 2:**
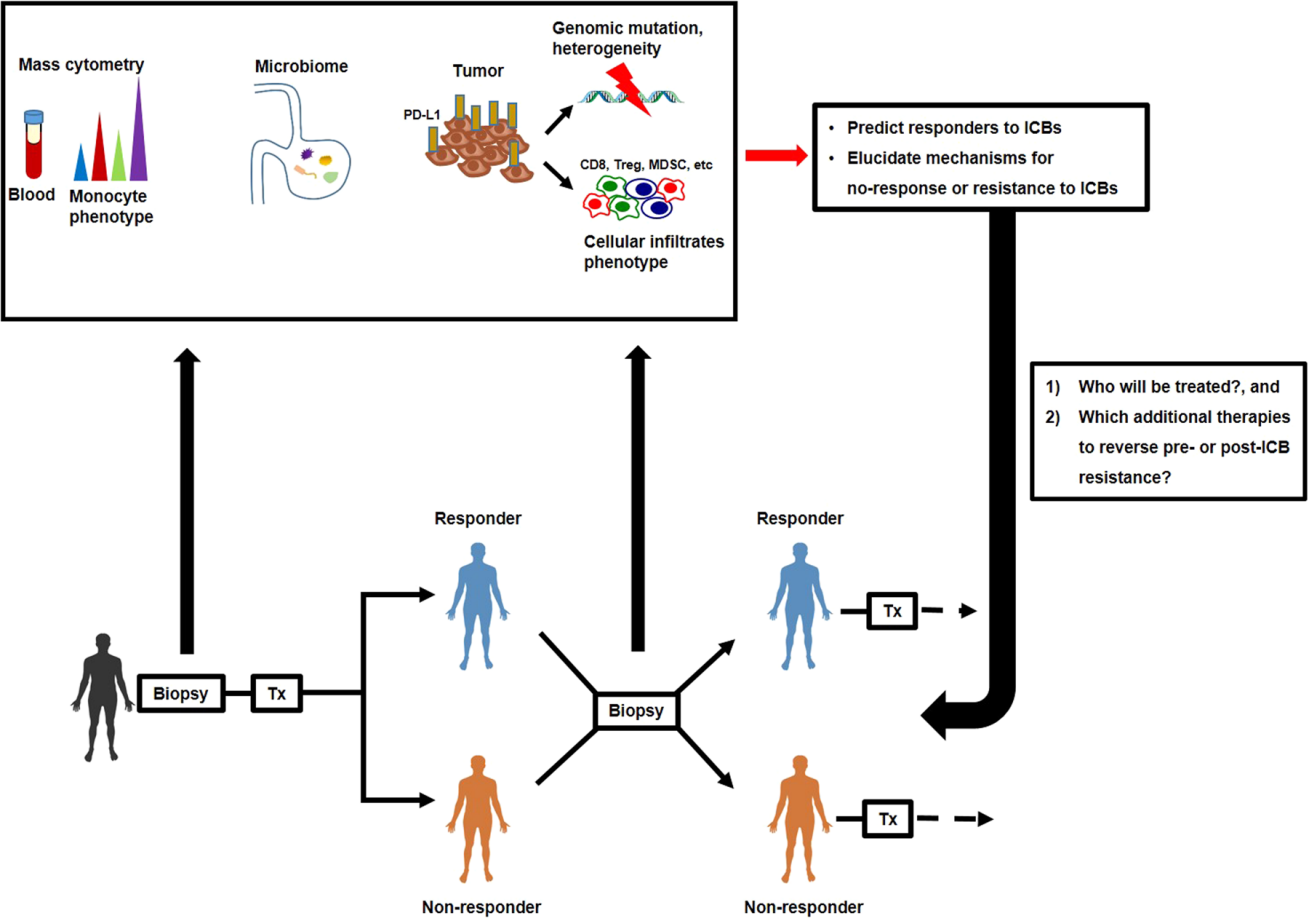
Prediction of the efficacy of ICBs based on biomarkers identified from biopsies at each time point. Several longitudinal analyses on genomic and immunologic signatures in biopsies (tissue or blood) of tumor patients pre- or post-ICB treatment suggest novel biomarkers for discriminating responders and non-responders. If patients are predicted to be a non-responder before or after ICB treatment, clinicians can, on an individual basis, determine whether additional therapeutic medications should be applied to resolve resistance associated with poor prognosis

Despite the remarkable success of ICBs in improving objective response rates in a subset of patients, it has been demonstrated that only ≤ 20–30% of tumor patients with NSCLC, RCC, and melanoma benefited from CTLA-4 or PD-1 blockade^17,23,48–50^. This unresponsiveness to ICBs can be identified in two types of patients: patients who did not respond at all (primary resistance), and patients who relapsed after a partial response to ICBs (acquired resistance)^51^. These non-responder patients endure high treatment costs and toxicities with little benefit from the treatments. To sustain the success that ICBs have achieved in the treatment of various tumors in clinical settings, specific prognostic indicators should be identified to predict whether a patient would be rescued by ICB treatment (Fig. 2).

### Cellular composition/characteristics of the tumor environment

Initially, PD-L1 expression in the tumor environment seemed to be positively correlated with response to PD-1/PD-L1-blocking antibodies, but several exceptions have led to the recognition of PD-L1 as an imperfect marker. However, several groups have endeavored to identify robust biomarkers for the purpose of discriminating responders from non-responders through genomic and immunologic analyses from biopsies before or after treatment. In a study for pembrolizumab in patients with advanced melanoma, CD8 T-cell density was analyzed by immunohistochemistry (IHC) in some tumor compartments^52^. When analyzing pre-treatment samples, patients with a good response had higher CD8 T-cell density in the invasive tumor margin compared with specimens from patients with progressive disease. Moreover, there was a significant correlation between the proximity of PD-1/PD-L1 expressing cells and favorable prognosis after therapy, indicating that physical accessibility between PD-1^+^, possibly CD8 T cells, and PD-L1^+^ cells would be the pre-requisite for an effective PD-1 blockade. Based on these observations, a predictive model for a clinical response was established. To test this model, pre-treatment specimens from 15 patients treated with pembrolizumab were blindly examined. Consequently, 9 out of 9 patients who experienced a favorable response and 4 out of 5 patients with progressive disease were accurately predicted, while one patient with stable disease was predicted to show a response to the therapy^52^. To identify robust predictors further, an in-depth longitudinal analysis for tumor samples was performed^53^. This study cohort consisted of 53 metastatic melanoma patients initially treated with ipilimumab, followed by a treatment of pembrolizumab for non-responders. Specimens were obtained before ipilimumab treatment, while on-treatment, and after a clinical response was evaluated (response vs. progression). Non-responders were treated with pembrolizumab, and biopsies were obtained at early and late stages of treatment. Among those samples, analysis of biopsies collected immediately after CTLA-4 or PD-1 blockade treatment provided highly correlated predictors for response rates. In an immunological assay, the density of CD8^+^, CD4^+^, CD3^+^CD45RO^+^ PD-1^+^ or PD-L1^+^ cells was higher in responders compared to non-responders. The proximity between CD68^+^ myeloid cells and CD8^+^ T cells was also higher in responders, though not significantly. In addition, a recent study has demonstrated that peripheral blood mononuclear cell (PBMC) immuno-profiling could predict response rates in melanoma patients treated with nivolumab or pembrolizumab. Using high-dimensional single-cell mass cytometry, the authors searched for differential signatures in PBMCs from responders vs. non-responders^54^. Among alterations, the increase in classical monocyte (CD14^+^CD16^−^CD33^+^HLA-DR^+^) frequency was the most prominent in responders, indicating that biomarkers from blood sample collections, rather than those from invasive tumor samples, can be used in clinical practice (Fig. 2).

### Genomic/transcriptomic analysis reveals the difference in responder vs. non-responder patients

Given that the magnificent efficacy of immunotherapy was demonstrated in human studies, cytotoxic T cells work properly when they recognize antigen loaded onto MHC molecules on the surface of tumor cells. Because T cells are selected to maintain central tolerance in the thymus, these antigens would be the mutated forms of self-antigens, so-called neoantigens, created by tumor-specific chromosomal alterations and viral genomic substances. In this context, the efficacy of ICBs has been markedly outstanding in patients with highly mutated tumor burdens^55^. In 2014, whole-exome sequencing analysis on tumor samples from ipilimumab or tremelimumab-treated patients identified that the mutational load was directly correlated with a clinical benefit in advanced-melanoma patients^56^. Of note, the neoantigen landscape was also elucidated in patients with a strong response, and the peptides derived from these neoantigens activated T cells from patients ex vivo. Furthermore, a similar tendency in the circumstances of PD-1 blockade was identified. NSCLC patients treated with pembrolizumab were analyzed for tumor DNA sequencing^57^. The patients with significantly enhanced clinical efficacy were imprinted with an elevated nonsynonymous mutation load. Among them, the signatures related to smoking-induced mutation, neoantigen burdens, and DNA repair mutation were linked to a favorable clinical efficacy.

The genetic alteration in tumor cells has been attributed to defects in the DNA-related machinery. In particular, mutations in mismatch repair (MMR) proteins significantly enhanced the error rate in tumor cells, which can result in abnormal DNA microsatellites^58^. This microsatellite instability (MSI) is associated with the response of ICBs. In a phase 2 clinical study for pembrolizumab-treated patients with progressive metastatic colorectal carcinoma, the rate of the objective response was higher in patients with mismatch repair-deficient tumors than in patients with mismatch repair-proficient tumors^59^. A total of 1782 somatic mutations were detected in tumors with a mismatch repair deficiency, while only 73 mutations were identified in mismatch repair-proficient tumors, and higher mutation loads were associated with longer progression-free survival. Furthermore, a study indicated that not only colorectal cancer but also 12 different types of tumors harboring MMR deficiency were sensitive to PD-1 blockade^60^. Interestingly, there are several patients with high mutation loads who do not respond to ICBs^61,62^. This might be the consequence of intratumor heterogeneity, as multiregional genetic analysis of tumors from metastatic RCC patients revealed that spatially separated tumors exhibited discordant somatic mutation patterns^63^. In NSCLC and melanoma patients, sensitivity to pembrolizumab and ipilimumab was apparent in patients with high neoantigen loads and low heterogeneity in their tumor^64^, indicating that tumor clonal variability should be considered as a biomarker for the ICBs-responder prediction (Fig. 2).

In addition, genes for T-cell activation, antigen presentation, IFN-γ-related subunits, cytolytic markers, among others, were upregulated in samples collected from responders while on-treatment^65,66^, suggesting that these characteristics could be utilized as indicators to predict response rates. In contrast, the expression of a few genes, such as vascular endothelial growth factor (VEGF), was lower in responders in comparison with non-responders, arguing that the resistance mechanism could be a target for combination therapy, as other have suggested^67,68^. In a consecutive study, whole-exome sequencing was performed on the biopsies from the same cohort of metastatic melanoma patients treated with sequential ICBs to complement the previous study^69^. In patients resistant to single or double ICBs, there was a high burden of copy number loss in chromosomes in which various tumor suppressor genes (*FOXO3*, *PRDM1*, *PTEN*, *FAS*, etc.) were located. Although more efforts are needed to expand the cohort size and to broaden these criteria, this study provides an important guide on how to manage the ICB regimen. The inspection of biopsies obtained at each time point provides several mechanisms by which the efficacy of ICBs is restricted (Fig. 2). Regulating those inhibitory circuits with serial ICB treatment could relieve patients of clinical resistance, which we discuss below.

### The intestinal microbiome composition

Several studies from independent groups have shown that gut microbiota is required for the therapeutic effects of ICBs. These experiments conducted in mice have shown that the intestinal microbial composition determines how they respond to chemotherapy and ICBs, presumably mediated by the induction of DC maturation and Th1 responses in the tumor environment^70,71^. Experimental data supporting the hypothesis on the correlation between the colon microbiome and the clinical response to ICBs have been recently reported by multiple investigators^72–74^. For instance, three studies comparatively analyzed the bacterial families, species or diversity in stool by a metagenomic approach using whole-genome sequencing of 16S ribosomal RNA to investigate the microbiome of NSCLC, RCC^72^, and metastatic melanoma patients^73,74^ treated with anti-PD-1 immunotherapy. These studies showed that patients with a high diversity of microorganisms and with specific species (e.g., *Ruminococci, Bifidobacteria*, and *Enterococci*) exhibited a favorable response to PD-1 blockade. To clarify the causality between ICBs efficacy and specific commensal species, germ-free mice were recolonized with the bacteria isolated from patient stool using fecal microbiota transplantation (FMS). Mice transplanted with stool from responders exhibited significant regression of tumor growth compared to those receiving stool from non-responders. In addition, microbiota composition in melanoma patients treated with ipilimumab was analyzed to distinguish specific bacteria associated with the efficacy and adverse effects, such as colitis^75^. This study revealed that the *Faecalibacterium* genus and other Firmicutes were responsible for the prolonged survival of patients and the occurrence of ICB-mediated-colitis. Although more investigations should be performed to confirm the relation between the microbiota and the clinical response, oncologists may need to consider the proper use of anti- or probiotics before and during ICB treatments to maximize the therapeutic effect (Fig. 2).

## Resistance mechanisms to ICBs: future targets for combinatorial therapy

Many efforts have been made to gain mechanistic insights into the wide spectrum of patients who exhibit primary and/or acquired resistance to ICB and to find ways to maximize efficacy/coverage via combinational strategies. The approximately 430 and 390 on-going clinical studies investigating combination therapies with pembrolizumab and nivolumab, respectively, exemplify the sheer volume of efforts and resources invested in finding a more personalized, combinatory regimen. The reasons as to why some patients do not benefit from ICBs are largely associated with the defects in the context of T-cell behavior within the tumor environment. As such, many combination strategies target other biological pathways to better induce longer-lasting T-cell activity within the tumor environment. To properly induce tumor-specific T-cell responses, tumor antigens should be engulfed by professional antigen-presenting cells such as dendritic cells (DCs). Then, inflammatory stimuli activate DCs to migrate into adjacent lymph nodes in which T cells are educated by DCs presenting tumor antigens. The differentiation of tumor-specific T cells into effector T cells crucially contributes to tumor immunosurveillance. The interaction between CD28 and CD80/86 is indispensable for fully activating T-cell functions, while PD-1 and CTLA-4 blunt their ligation. Activated tumor-specific T cells differentiate into effector cells and then home to the tumor tissues attracted by chemokines and attack tumor cells expressing antigens loaded onto MHC molecules. Though ICBs potentiate T-cell activation, the other steps of immune-induced tumor-killing should be operated precisely to control tumor growth. In the following sections, we introduce the mechanisms of how both tumor-intrinsic and -extrinsic factors regulate immune activation cycles to restrict ICB efficacy.

### Insights into primary resistance and non-responders to ICBs

Approximately 40 to 60% of melanoma patients treated with nivolumab, as well as over 70% of patients treated with ipilimumab, show primary resistance^9,17,44,76,77^. IPRES (Innate anti-PD-1 Resistance Signatures) describe a set of genes inherent to the patient that have been attributed to the primary mechanism of resistance to PD-1 blockade^66^. Comparative transcriptome analysis between melanoma and pancreatic patients who do and do not respond to PD-1 blockade revealed that non-responders showed an enrichment of genes associated with mesenchymal transition, wound healing, and angiogenesis^66^. In another aspect, other genomic indicators of poor immunogenicity, such as epigenetic downregulation of chemokines (lower T-cell recruitment), upregulation of endothelin receptors (higher tumor angiogenesis and survival), MHC downregulation and low neoantigen load (lower antigen exposure), and impaired DC function (lower antigen presentation), have been described and attributed to what is popularly termed “cold” tumor types^78,79^. Therapeutic interventions to improve the responsiveness of these “cold tumors” to ICB are being investigated with various combinational therapies in clinical trials.

### Tumor-intrinsic factors related to resistance

**Fig. 3:**
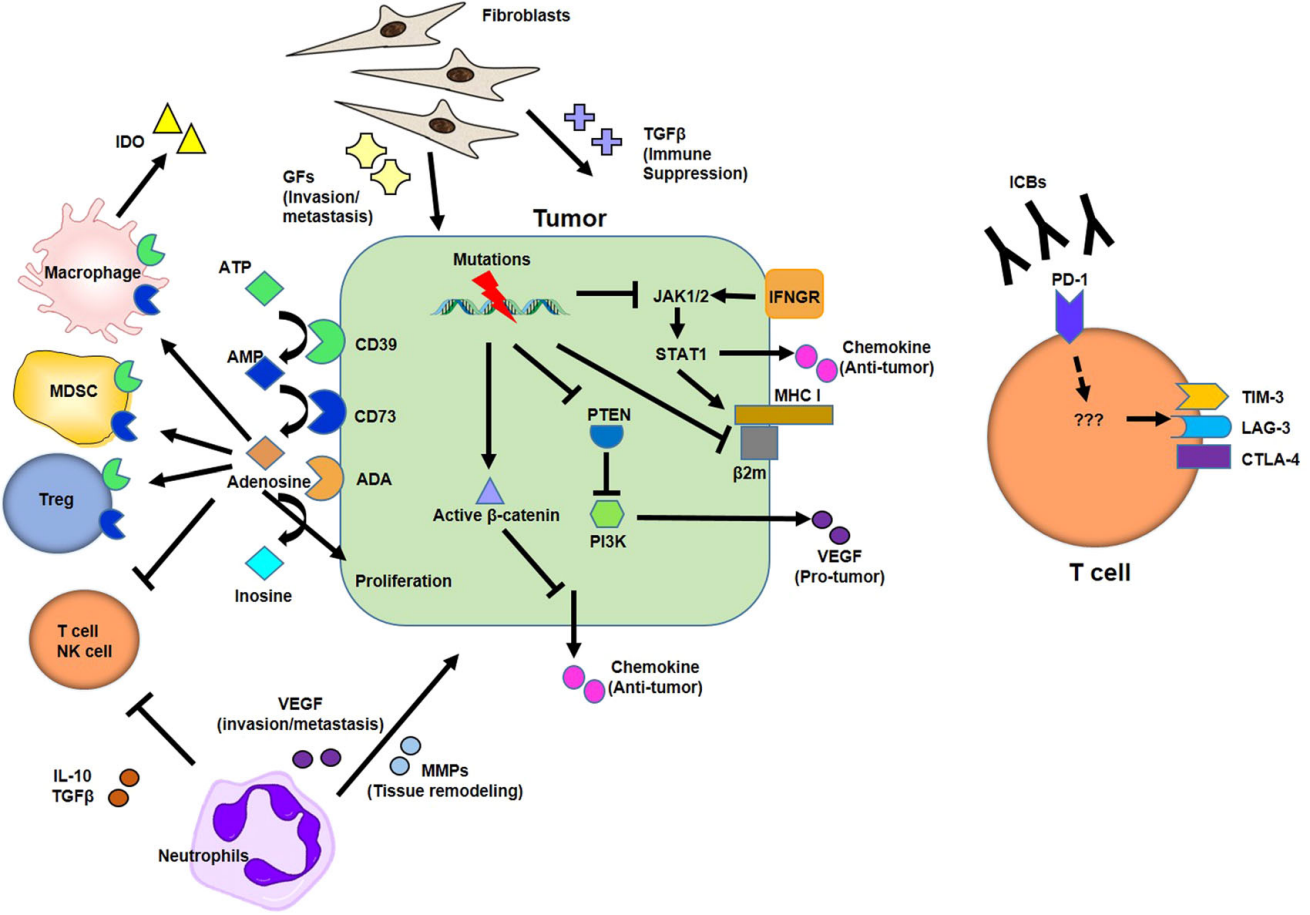
Tumor-intrinsic and -extrinsic resistance mechanisms to ICBs. Tumor-intrinsic resistance mainly originates from gain- and loss-of-mutations in oncogenes and tumor suppressor genes, respectively. Mutations resulting in JAK1/2 malfunction break IFN-γ signaling pathways important for chemokine production and MHC I expression. PTEN loss mediates constitutive activation of PI3K to produce VEGF. The expression of MHC I and β2-microbulin (B2M) is also downregulated in patients with no-response to ICBs. Gain-of-function of β-catenin inhibits chemokine expression. In T cells, on the other hand, PD-1-blockade induces alternative TIM-3 (not significantly LAG-3 and CTLA-4) expression to limit activation. ATP metabolites participate in raising the tumor suppressive environment. Tumor suppressive myeloid cells and Treg cells express high levels of CD39 and CD73, degrading ATP and AMP into AMP and adenosine, respectively. Adenosine inhibits effector cell activation while promoting tumor cell proliferation. Adenosine deaminase (ADA) expressed on tumor cells degrades adenosine into inosine. Tumor-associated neutrophils promote tumor metastasis via VEGF/MMPs secretion while anti-tumor effector cells are subverted by IL-10/TGF-β. In addition, tumor-associated fibroblasts also secrete various metastatic/immunosuppressive mediators, which can exacerbate tumor progression

As mentioned above, tumors with higher initial mutational burdens appeared to be positively correlated with therapeutic outcomes. However, the mutations in tumor cells do not always guarantee a favorable response to ICBs, as intratumoral heterogeneity and mutations that might be advantageous for immune-escape and survival can be another manifestation of the high mutational burden^80^. Loss of function mutations in Janus kinases JAK1 and JAK2 were observed in patients who exhibited no-response to pembrolizumab^81^ (Fig. 3). IFN-γ released by T cells post ICB was shown to sensitize tumor cells to activate these tyrosine kinases, after which tumor cells secrete T-cell-attracting chemokines and upregulate PD-L1 expression^82^. Similarly, melanoma patients with defects in the IFN-γ signaling pathway were resistant to ipilimumab treatment^83^. Tumor growth was also uncontrolled in mice bearing tumors with low IFNGR1 expression. Interestingly, chronic IFN-γ exposure rendered tumor cells resistant to ICBs through epigenome/transcriptome changes driven by JAK/STAT1, which is independent of PD-L1 expression^84^. CDK5, a cell-cycle regulator, has been shown to modulate and enhance PD-L1 expression in brain cancers in response to IFN-γ exposure^85^. Furthermore, CDK5 disruption resulted in enhanced expression of interferon response factors, indicating the tight epigenetic regulation that exists between the components of the cell-cycle and IFN signaling pathways. In this regard, IFN-γ release can be seen as a ‘‘double-edged sword’’, where acute IFN-γ is beneficial to the initial T-cell-mediated anti-tumor efforts (immune cell recruitment and activation) as well as in inducing MHC expression on tumor cells, whereas chronic IFN-γ exposure induces further mutations in tumor cells and leads to PD-L1 upregulation^84^.

Oncogenic signals are also responsible for resistance to ICBs. In a subset of melanoma patients, active β-catenin levels were detected, along with the absence of T cells and CD103^+^ DCs in tumor tissues. β-catenin suppressed CCL4 secretion, which is important for the recruitment of T cells/DCs into the tumor bed (Fig. 3). The same molecular phenomenon has also resulted in resistance to PD-L1/CTLA-4 blocking antibodies in mouse experimental models^86^.

Gain-of-function mutations in BRAF, which drive MAPK pathway activation, have been demonstrated to potentially regulate the efficacy of immunotherapies. BRAF promoted immunosuppressive IL-1 secretion in the stromal cells of melanoma patients^87^. In addition, BRAF inhibited the expression of melanoma antigen, such as MART-1. BRAF or BRAF/MAPK inhibitor therapy enhanced anti-tumor immune responses in melanoma patients. However, an increase in PD-1 or PD-L1 was observed in patients with resistance to those inhibitors. In this case, additional ICB treatment may improve the therapeutic effect of BRAF/MAPK inhibitors^88^. Phosphatase and tensin homolog (PTEN) tumor suppressor loss has also been attributed to the resistance to PD-1 blockade in a mouse tumor model. The PI3K pathway was activated in the absence of PTEN, promoting immunosuppressive CCL2 and VEGF secretion. Tumor growth significantly regressed in the ICBs and PI3K inhibitor co-treatment group^89^ (Fig. 3). Concurrently, PTEN loss in a human melanoma dataset correlated with lower gene expression of IFN and reduced CD8^+^ T-cell infiltration^89^. In metastatic uterine leiomyosarcoma, biallelic PTEN loss and changes in neoantigen expressions were correlated with resistance to pembrolizumab monotherapy^90^.

Alternatively, cancer cells may have defective β-2-microglobulin and HLA class I functions, leading to abnormal tumor antigen processing and presentation, respectively, facilitating escape from immune-surveillance^91–93^. Tumor cells also repressed the expression of Th1-type chemokines by epigenetic regulation, resulting in tumor cells restricting T cells from entering the tumor environment via negative regulation of proliferation, viability, and migration of tumor-specific T cells^94,95^.

### Tumor-extrinsic factors related to resistance

It is now commonly accepted that cancer cells constitute suppressive networks with surrounding stromal cells and immune cells^96^. Typically, the tumor environment evades immune-mediated eradication through depleting essential nutrition or producing deleterious materials for immune cells. Cancer cells and myeloid cells express indoleamine-2,3-dioxygenase (IDO) to catalyze tryptophan into kynurenine, which induces T-cell dysfunction due to the deficiency of essential amino acid^97^. Furthermore, IDO enhances MDSCs and Treg cell recruitment into the tumor microenvironment^98,99^. IDO inhibitors have been shown to potentiate anti-tumor effects in combination with immune checkpoint blockers^98,100^ and are on the brink of being approved in clinical trials. Adenosine, also abundant in the tumor environment, inhibits effector functions of NK cells/CD8 T cells and induces suppressive M2 macrophage differentiation and myeloid-derived suppressor cell (MDSC) accumulation.

Adenosine also promotes the proliferation of cancer/blood vessel endothelial cells to broaden the tumoral niche^101–103^. Two ectonucleotidases, CD39 and CD73, have critical roles in generating adenosine. In inflammation, ATP released from cancer or immune cells is converted by CD39 into AMP, which CD73 then metabolizes into adenosine^104^ (Fig. 3). A recent study has demonstrated that a CD73-blocking antibody increases the anti-tumor effect of CTLA-4/PD-1 inhibition, suggesting that the combination of such treatments could be beneficial in clinical settings^105^.

Accumulating evidence indicates that MDSC infiltration in tumor tissues is strongly attributed to poor prognosis in ICB-treated patients due to the suppressive activity of the tumor-specific T-cell response^106^. In an analysis of peripheral blood mononuclear cells (PBMCs) of metastatic melanoma patients, MDSCs were higher in non-responders compared with responders during ipilimumab therapy, indicating discriminative biomarkers for the ICB response^107^. Recently, an attempt to overcome MDSC suppression by targeting the gamma isoform of phosphoinositide 3-kinase (PI3Kγ) has been introduced^108^. A PI3Kγ inhibitor converted MDSCs into immunogenic myeloid cells, and subsequent tumor-specific CTL responses were enhanced in combination with ICBs. Other myeloid compartments, such as tumor-associated macrophages (TAMs) and neutrophils (TANs), have been shown to contribute to ICB resistance in distinct ways. TAMs have been primarily known to induce angiogenesis and enhance tumor survival via immunosuppressive factors and degradative proteins, thus facilitating therapeutic resistance^109^. Furthermore, recent in vivo imaging analysis revealed that PD-1^-^ macrophages engulf anti-PD-1 antibodies that bind to PD-1^+^ CD8^+^ T cells in a FcγR-dependent manner to disrupt PD-1 efficacy^110^.

TANs are known to also release secretory factors, such as MMP2 and hepatocyte growth factor (HGF), to induce ECM remodeling and recruit other immunosuppressive immune cells that aid in cancer immune-escape^111^. In a mouse model of KRAS-mutant lung tumor, pro-inflammatory IL-17A cytokine secretion has been shown to recruit neutrophils to the tumor sites, and anti-Ly6G depletion of neutrophils was found to be more effective than PD-1 blockade in treatment of the tumor^112^. Positive correlation between KRAS mutation and IL-17 levels was also found in lung cancer patients^112^. In an analysis of 720 advanced-melanoma patients treated with ipilimumab, a higher neutrophil baseline and neutrophil-to-lymphocyte ratio in the blood were found to be significantly associated with lower survival rates^113^. Taken together, TANs seem to contribute to resistance against ICBs.

TGF-β is another powerful negative regulator of effector T cells that is actively exploited by various compartments of the TME. A recent study of patients with metastatic bladder cancer who did not respond to atezolizumab therapy has shown that tumor-associated fibroblast- and collagen-rich extracellular matrices upregulate TGF-β expression, which could inhibit CD8^+^ T-cell infiltration into the tumor environment^114^. Combination of ICBs with TGF-β blockers proved to be synergistic in tumor mouse models

PD-1-blocking antibody therapy itself also induces the expression of inhibitory molecules in CD4/CD8 T cells to evade attack from immune cells. An increase in TIM-3 was found in T cells from patients with non-small cell lung cancer not responding to PD-1 blockade^115^. In a mouse model, therapeutic sensitivity to PD-1 blockade was restored in combination with a TIM-3-blocking antibody injection (Fig. 3). In addition, Treg infiltration into tumor tissues reduces the effector T-cell/Treg ratio, which is a negative indicator in tumor patients^116^. Treg depletion using Fc-optimized anti-CD25 antibody induced a synergistic and therapeutic effect with either nivolumab or pembrolizumab in an established tumor model^117^.

On the other hand, epigenetic studies have revealed that although ICBs do lead to transcriptional reprogramming within the exhausted T-cell pool, they induce minimal development in genes associated with memory T-cell generation^118^. Moreover, exhausted T cells displayed extensive changes in accessible chromatin, changes that were dissimilar to those of their effector T-cell counterparts^119^. Exhaustion-specific enhancers in exhausted T cells showed distinct motifs at RAR, T-bet, and Sox3 signature transcription factor binding sites^119^. The magnitude difference in the profile of regulatory regions between exhausted and functional CD8 T cells (44.48% of all chromatin-accessible regions differentially present) were greater than those observed in gene expression (only 9.75% differentially expressed genes), suggestive of a large rewiring of accessible chromatin networks associated with the exhaustion state. Thus, co-treatment with epigenetic and/or metabolic modulators has been proposed to overcome the inherent limitations of ICBs^120^.

## Conclusion

The cancer field in the past few years has witnessed a great advancement in the comprehension of immune-surveillance in the tumor environment. Along the way, researchers have assumed that the regulation of immune checkpoint pathways might have an impact on anti-tumor immunity, thereby leading to the successful administration of CTLA-4/PD-1-blocking antibodies to treat many types of cancers. However, only a minority of patients’ tumors were reduced by this treatment due to tumor-intrinsic or -extrinsic resistance mechanisms, necessitating a clarification of genomic, epigenomic, transcriptomic, as well as cellular features linked with tumor response and resistance. Using novel molecular and diagnostic technologies, the resistance mechanisms should be fully addressed to offer custom-made therapies or optimal therapeutic combination to potentiate clinical responses for patients with a variety of tumors.

In other aspects, several attempts have been made to stimulate anti-tumor activity and/or to eliminate immune-threatening suppressors: the development of agonistic antibodies against costimulatory receptors (ICOS, GITR, 4-1BB, etc.)^121,122^, therapeutic vaccines that induce immune responses to tumor-specific antigen^123,124^, and agents that remove suppressive myeloid cells (MDSC, M2 macrophage, tolerogenic DC, etc.)^125,126^.

Importantly, recent breakthrough discoveries have demonstrated that some species of microbiota could manipulate anti-tumor immune responses to give rise to favorable clinical responses through the induction of DC maturation and a Th1 response, indicating that probiotics or antibiotics-mediated microbial rearrangement perhaps stimulate effective immune responses^70,71^. Considering the conditions of cellular, genomic, and microbial status within individual patients, strategies mentioned above will be integrated optimally to augment the therapeutic effect, which could, in the near future, save the lives of patients suffering from uncontrolled malignancies.
